# Smoking at School: Views of Turkish University Students

**DOI:** 10.3390/ijerph6010036

**Published:** 2008-12-23

**Authors:** Nazmiye Erdogan, Irfan Erdogan

**Affiliations:** 1 Social Science Vocational School, Baskent University, Ankara, Turkey; 2 Faculty of Communication, Gazi University, Emek, Ankara, Turkey; E-mail: irfan.erdogan@gazi.edu.tr

**Keywords:** Student cigarette use, cigarette regulation, university smoking policy, university students, student views, designing nonsmoking environment, cessation programs

## Abstract

The recent interest in cigarette smoking among university students has brought attention to problems concerning opinions, attitudes, prevention, health education, policy formulation and implementation. This survey research tested five hypotheses on the views of college students about smoking in school hallways and cafeteria, compliance with anti smoking laws, considering cigarette smoking as an expression of freedom of choice, teachers’ smoking in classrooms and in their offices, and school administration’s policy on enforcing the law. Hypothesized differences between students’ views on the issues according to gender, smoking status and years at school were investigated. Data were obtained from 3,659 students attending six universities in Ankara, Turkey. The study findings provided support for all the hypothesized differences (except a single issue). Males and females differed significantly on all the issues studied. The majority of nonsmoking students have anti-smoking views in regards of the studied issues as compared to regular and occasional smokers. Smokers and nonsmokers markedly disagree on banning cigarette smoking in the cafeteria and hallways. However, the majority of students are against teachers’ smoking in classrooms and in their offices with the doors open. Although most students want a smoke free environment, there is no active-anti smoking policy on smoking by universities. Findings point out the need for campus-wide effective smoking prevention programs, as well as cessation programs and services for the students.

## 1. Introduction

Cigarette use of the university students and administrative policies concerning smoking are significant aspect of critical public health issues. New policy initiatives and environmental design types have emerged along with anti-smoking policies at universities all over the world, especially since the 1980s. School administrators, interior and exterior landscape designers, and other responsible parties started anti-smoking programs and excluding elements in the school environment that promote smoking. On the other hand, despite the widespread social communications and findings about the consequences of smoking that portray a highly objectionable human condition and a dreadful end for smokers and secondhand smokers, as Stockdale *et al*. [1] indicate, the recent evidence shows an increase in smoking rates, especially among adults aged 18 to 25. Smoking is still the single biggest preventable cause of death. According to the World Health Organization statistics, smoking claims 5.4 million lives each year and cigarette consumption has reached epidemic proportions globally. Over 15 billion cigarettes are smoked worldwide everyday. Every eight seconds, someone dies from smoking [2]. Turkey has achieved some progress in reducing tobacco consumption: The proportion of daily smokers among adults had decreased from 43.6% in 1989 to 32.1% in 2003, yet the smoking rate among adults remains much higher than the OECD (Organisation for Economic Co-operation and Development) average of 23.7% [3].

Recent studies demonstrate that smoking among college students all over the world is on the increase [4–8]. For instance, several studies among college students in the U.S. have found continued high rates of smoking, with almost 30% of students reporting smoking [9]. Similar findings were obtained in Beirut [10, 11]. Moran *et al*. [12] and Nichter *et al*. [13] found that the college years have been characterized as a time of increased risk to smoking initiation as well as movement from intermittent or social smoking to more regular patterns of use. The visibility of smoking on campus, the lack of restrictions on smoking, the presence of social imitation, and the ease of purchasing cigarette are also crucial factors in smoking. Most studies have established the strong link between social influences and the behavior of smoking [5, 14, 15], between peers who smoke or peers who pressure others to smoke and the smoking status of an individual [1, 16–19]. Supporting the findings of similar studies, Harakeh *et al*. [20] found that smoking of the best friend influenced smoking of the younger sibling. Furthermore, these social influences were stronger among those who reported starting or increasing their smoking since coming to the university than among those who maintained or decreased their level of smoking. Focusing on the college student opinions of no-smoking policies, some studies found that nonsmoking students have the most favorable attitudes toward no smoking. Male students who are current smokers have the least favorable attitudes. The lowest level of agreement by all subgroups is provided for prohibiting smoking everywhere on campus [21]. More non-smokers support smoking ban in their school than smokers do and more smokers support “restrictions” than non-smokers do [22, 23].

The rise in cigarette smoking among students is a growing public health, education and social relations concern, especially in developing countries wherein efforts to prevent smoking are severely lacking. Turkey has the highest smoking rate in Europe and the third highest rate in the world [6, 24, 25]. Cigarette, like drinking tea and coffee, is an integrated part of the daily life and socializing experience in Turkey. In a nationwide survey conducted in 2002, it was found that the prevalence of smoking among 15 year-olds and above was 45.3% among males and 18.3% among females. According to findings of other studies in Turkey, the prevalence of smoking ranges between 30% and 63% [26–30]. Smoking rate tends to increase in the universities. For example, Senol and colleagues [31] observed that 22% of medical students were smoking in the first year of study and this rate had risen to 27% by the sixth year. Namely, one-third (32.3%) of original nonsmokers in the first year had also become smokers by the end of the sixth year. Similarly, Aslan *et al*. [29] found that the smoking prevalence among last year university students was significantly higher (49.8%) than for first year students (34.0%).

Some studies indicate that there are outstanding levels of compliance with smoking control laws, especially at the U.S. universities. Contrarily, some studies found that the rapid growth in various laws restricting or banning smoking has not caused much of a backlash among smokers in the developed countries [32]. An irrational and illogical opposition against any intervention on smoking and widespread groundless justifications such as “reversed rationalism” [33] can be found among people, including university students and university teachers in Turkey.

Findings of the related literature discussed above also suggest that there is a continuous need for research, especially in countries like Turkey. The rationale of this study is based on the regrettable fact that there is a widespread lack of interest of responsible parties on the human health and establishing a smoke-free university environment. Based on such rationale, this study aims at providing fresh information and discussion in order to contribute to the accumulated knowledge about the cigarette smoking and problems related with prevention policies. Such information is helpful in developing comprehensive, effective and culturally relevant cessation programs. It also aims at reminding all involved parties that they should pay proper attention to the urgency and magnitude of the problem and should act to initiate appropriate prevention and intervention programs. Keeping this objective in mind, this study focused on the opinions of Turkish university students on smoking at school.

Based on the problems discussed above, the study extracted five hypotheses to test:

H_1_: There are gender differences about the issues under study.

Gender itself cannot be a cause of smoking, but the way of students’ acculturation in a society can create significant differences. Females and males have their own reasons for smoking or not smoking. These reasons are part of the justification of their behavior as well as reflections of prevailing socio-cultural structure. Despite the fact that some of these reasons are similar for both sexes and others are not, it is expected that there are differences between sexes in terms of their views on issues about smoking. These differences are enhanced by the Turkish female culture that has a general thoughtfulness and interpersonal sensitivity.

H_2_: There is a relationship between the smoking status and the views about issues studied.

Individuals provide explanations to justify their behavior to themselves and others. They seek for congruency between their actions and thoughts. Therefore, it is expected that regular smokers, occasional smokers and nonsmokers hold and express opinions that reinforce their smoking behavior. This calls for the hypothesis that there are differences among regular smokers, occasional smokers and nonsmokers about the issues questioned.

H_3_: There is positive relationship between the years at school and regular smoking.

The previous research findings imply that Turkish university environment cultivates relations and feelings that promote smoking more than nonsmoking. Thus, it was hypothesized that more cigarette smokers would be found among the senior students as compared to the new comers. This means that more students will start smoking than quitting during their university years, despite the negative relationship generally assumed between education and smoking.

H_4_: There is no relationship between the years at school and the views about issues questioned.

The above rationale also calls for the hypothesis that there would be no significant relationship between school status (years at school) and students’ views on the issues studied. The factors like quality of life, mental processes and formal and informal education are functions and integral parts of the general intellectual level and behavioral mode in a society. In Turkey, it is usual to hear statements like “I have been smoking fifty years and nothing happened to me.” These kinds of statements are also uncritically reproduced by the mass media.

H_5_: There is a relationship between smoking status and the considering smoking as the expression of freedom of choice.

It is expected that smokers generally tend to reflect the ideas promoted by the proponents of the cigarette industry in their daily discourses in order to justify smoking behavior (selective exposure). Doing so, more smokers than nonsmokers are likely to claim that smoking is an expression of freedom of choice. Hence, it was hypothesized that there is a significant relationship between smoking status and considering smoking “as expression of individual freedom of choice.”

## 2. Method

### 2.1. Population,Sample and Data Collection

There are four state and six private universities in Ankara, with 196,135 students in 2007–2008. The study population included 163,009 students from Ankara University (41547), Baskent University (8691), Gazi University (57834), Hacettepe University (29727), Middle East Technical University (22100) and Atilim University (3110) [35]. Due to financial and bureaucratic difficulties in drawing a sample frame, extracting representative samples and collecting data accordingly, a convenient sample of 3,659 students was drawn independently from each university (750 from Ankara University, 750 from Baskent University, 611 from Gazi University, 641 from Hacettepe University, 575 from Middle East Technical University and 332 from Atilim University). The sample size included 2.25 % of the study population. Student cafeterias and campus outdoors were used for data collection.

Data collection was done in March, April, May, October and November 2007, by means of self-administered one-page questionnaire. Everybody sitting in cafeterias and campus outdoors were given the questionnaires after short explanation about the survey. Every student accepted to fill up the questionnaire and handed back the questionnaire after completing it. However, small number of respondents did not provide answer to one or two questions. Such non-responders were less than one percent (ranging between 0.2 % and 0.7 %).

### 2.2. Variables and Measures

The questions focused on gender, university attended, school status, smoking status and students’ opinion about the following issues: (1) smoking in school hallways and student cafeteria, (2) complying with the cigarette law; (3) considering cigarette smoking as expression of freedom of choice, (4) school administration’s policy on enforcing the law, and (5) teachers’ smoking behavior.

School status was defined as the number of years at the university and categorized as 1 (first year), 2 (second year), 3 (third year) and 4 + (last year).

Smoking status was measured by a closed-ended question (do you smoke?) with three choices: yes (regular smokers), sometimes (occasional smokers) and no (nonsmokers).

The students’ opinions about the five issues were measured by asking them the following closed- ended questions that show their approval or disapproval:

1. Students’ smoking behavior: What do you think about cigarette smoking in the school hallways? What do you think about smoking in the student cafeteria?
2. Complying with cigarette law: Is it right to smoke indoors despite the cigarette law?
3. Smoking as freedom of choice: Is smoking an expression of individual freedom?
4. Law enforcement: Do your school administration should enforce the cigarette law or not?
5. Teachers’ smoking behavior: What do you think about those teachers who smoke at the classroom? What do you think about those teachers who smoke with their office door open?

### 2.3. Statistical Analyses

Univariate analyses were used in order to determine the nature of distribution of responses within each variable. Bivariate analyses were utilized to test the hypotheses and assess the statistical significance. Frequency distributions for univariate analyses and Chi-square for bivariate analyses were used since all variables were measured at either nominal or categorical level. Partial chi-square distribution and correlations were used for additional information. Level of significance for bivariate comparisons were set as 0,01. The entire questionnaire was pre-tested for face and content validity as well as comprehensibility in a pilot study before implementation. Cronbach’s alpha was used for the reliability test for the opinion measures.

## 3. Findings

Cronbach’s alpha test was used to test the reliability of measurement of student opinions. The result shows a high degree of reliability (α= 0.83).

### 3.1. General Characteristics

The study sample of 3,659 students were comprised of 28.5 % first year students, 23.4 % second year, 24.7 % third year and 23.5 % fourth year and above. One third of respondents (33.4 %) were regular smokers, 14.8 % were occasional smokers and 51.8 % were nonsmokers. The rate of regular smokers was a little over the general rate in Turkey (32.1 %) reported by the OECD [3].

Findings show that great majority (83.1 %) want to comply with the cigarette law and would like to see the universities enforce the law (75.9%). A considerable majority do not approve of smoking in the hallways (68.6 %) and cafeteria (53.6%). Most students do not want teachers to smoke in the classrooms (80.1%) and want them to shut their office doors while smoking (64.4%). Similarly, great majority (73.5%) do not consider smoking an expression of personal freedom. Despite the existence of unfavorable views about smoking and favorable opinions in general, there are significant cross-sectional differences.

### 3.2. Hypotheses

Hypothesis # 1: The findings support the hypothesis about the gender differences on the issues. Chi-square tests show significant differences of opinion between male and female students (Table 1).

Findings indicate that females tend to smoke less than males. Of 1,753 male students, 39.5 % are regular smokers, 14.9 % occasional smokers and 45.5 % nonsmokers. Of 1,901 females, 27.8 % smoke regularly, 14.7 % smoke occasionally and 57.5 % do not smoke.

More female students than male students reported that smoking should not be allowed in the hallways (73.2% vs. 63.5%) and cafeteria (57.3 vs. 49.7 %). The percentages of females who think that teachers should close their doors and should not smoke in the classroom were higher than for males (66.8 % vs. 61.9% and 85.0% vs. 74.7%).

Most females (86.7 %), followed by males (79.3 %) also think that they should comply with the cigarette law. More females than males (82.0 % and 69.2 %, respectively) are inclined to demand that the law should be enforced by school administrations. There is a considerable difference between females and males (22.1 % and 31.2 %) in terms of considering cigarette smoking as an expression of freedom of choice.

Hypothesis # 2: Findings of the study confirm the hypothesis about the difference among students on the studied issues (table 2).

Great differences in opinion emerged about smoking in the hallways and cafeteria. As it was expected, there is a significant difference between smokers and nonsmokers on smoking in student cafeteria. Most regular smokers (82.7 %) and half of the occasional smokers (51.5 %) approve of smoking in the cafeteria, while most nonsmokers (78.5 %) do not. Students feel like they can smoke there because it is a place for eating, drinking and smoking.

Likewise, it was expected that most nonsmokers and some smokers would not approve smoking in the hallways. Findings generally support this expectation: Most nonsmokers (89.8 %) do not approve of smoking in the hallways, while a majority of smokers (64.5 %) approve.

Students’ opinion about teachers’ smoking behavior differs significantly across the three groups. However, a great majority of nonsmokers (92.8 %) and occasional smokers (79.2 %), and a majority of regular smokers (60.8 %) do not approve of teachers smoking in the classrooms. Similarly, 79.5 % of nonsmokers, 60.7 % of occasional smokers and 42.8 % of regular smokers indicated that teachers should close their office doors while smoking.

As expected, nonsmokers respond significantly more favorably than do occasional and regular smokers on obeying the cigarette law. Smokers are less inclined to respect the law (63.0 %) than nonsmokers (95.3 %). In general, most students (75.9 %) are in favor of enforcement of the cigarette law by the university: 93.6 % of nonsmokers and 76.2 % of occasional smokers want the university administration enforce the law. This percent decreases to 48.1 % among smokers.

Findings on the first two hypotheses showed that opinions of students about the studied issues are related with the smoking status and gender. These results raise two questions: Do results change if (1) the smoking status and (2) gender are controlled? In order to answer these two questions two partial chi-square correlations were performed. Firstly, relationships between gender and the studied issues were tested by controlling for the smoking status. The statistical analyses showed significance levels ranging from 0.001 to 0.81. Since smoking is significantly more common among males, it is expected that the opinions on all the studied topics will be different between the male and female smokers. The results supported this expectation: There were statistically significant differences ranging from 0.001 to 0.05 on all issues, except the opinions about teachers who smoke their office doors open. Nonsmoker males and females significantly differed in four issues. Secondly, relationships between the smoking status and the studied issues were tested by controlling for the gender. Statistical results showed significant differences on all issues at 0.001 level. The both sexes differ in their opinions within their own gender category.

Statistically significant (and insignificant) frequency distributions show that more females than males have environmentally and socially sensitive opinions on every issue studied.

Hypothesis # 3: The hypothesized difference on smoking rate and school status (years at school) were supported by the statistical test results (Table 3). Findings reveal that 40.2 % of the last year students smoke regularly and this rate is 10.2 % higher than that of the first year students. Conversely, rate of first year nonsmokers is 11.4 % higher as compared to the last year nonsmokers. The change is striking among females: Regular smokers among the first year female students are 23.6 % whereas it is 34.6 % percent among the fourth year students. The difference between the first year and the last year among males is less than females (8.1 % and 11.0 %, respectively). However, percent of regular smokers among male students are higher than females (37.3 % for the first and 45.4 % for the last years). More than one third of males (39.6 %) and one fourth of females (27.8 %) smoke regularly.

Hypothesis # 4: Tests comparing the school status (years at school) with the issues studied were not significant for the six issues out of seven at the 0.01 level (Table 4). The insignificant distributions according to the years at school show less than 2 percent differences on every issue studied. These small differences suggest the existence of a negative trend: Disapprovals lessen while approvals increase. There are more last year students than the first year students who approve the smoking indoors despite the law (20.1 % and 14.9 %) and teachers’ smoking in classroom (24.1 % and 19.5 %, respectively). These results indicate that years in school do not make any significant difference in students’ opinions in a positive sense.

Hypothesis # 5: (Table 2). There are significant differences of opinion among the three groups of students on the freedom issue. More smokers tend to see smoking as the freedom of individual choice: 40.6 % of regular smokers, 32.1 % of occasional smokers and 15.8 % of nonsmokers consider smoking expression of individual freedom (X2 = 242.60 df=2 p= 0,001). There are remarkable difference between the smokers and nonsmokers: Only 15.8 % of the nonsmokers consider smoking freedom of expression. This rate goes up to 40.6 % among the smokers. The rate is also considerably high among males (19.2 %, 35.0 % and 43.6 %) as compare to females (13.3 %, 29.4% and 36.5 %) on each category of the smoking status.

### 3.3. Other Findings

During the data collection, we observed that students have easy access to cigarette and freely smoke in cafeterias and restaurants in the universities. We did not observe any sign (other than few “no smoking” signs and posters) that is indicative of active anti-smoking campaign and/or cessation program.

We could not think of any reasonable rationale and, therefore, did not provide any hypothesis indicating whether there are significant differences between students attending the state and private universities. However, we found statistically significant differences between them on every item studied except two (considering smoking as freedom of choice and teachers’ smoking in the classroom). Our findings show that regular smokers from the two private universities were significantly higher than the state universities (39.4 % and 31.2 %, respectively). Students of private universities have significantly less anti-smoking view as compared to students of state universities. This finding disconfirm the findings of the studies that found lower SES as an independent risk factor for smoking initiation: Individuals with a higher SES have significantly lower odds for smoking initiation compared with individuals with a lower SES [36, 37]. Contrarily, the students from the private universities in Ankara smoke more and have less interest in health and environmental consequences of smoking. It seems that there is a need to investigate the causes of difference in Turkey and similar places.

## 4. Discussion and Conclusions

The rate of cigarette use (33.4%) could be seen as a positive sign. Actually, there should be very few smokers in the 21^st^ century that is regarded as the information and knowledge age. The study results support the findings of the previous studies that indicate that more non-smokers support smoking ban in their school than smokers do, and more smokers support “restrictions” than non-smokers [21–23]. It is a good sign to find out that most students have favorable opinions toward no smoking, law enforcement and smoke-free environment. However, findings also imply that especially smokers demonstrate less concern on social and environmental consequences of smoking.

Our observations during our lifelong experiences show that they do not smoke only if an authority forces them so. They stick to what is personally relevant to them in order to justify smoking and tend to show less sensitivity toward their own health, others and environment. They also enjoy breaking the law, if it is not enforced properly which is the prevailing case in Turkey. These observations and finding support the findings of studies showing that daily smoking is associated with increased reactivity and decreased emotional stability [e.g., 38]. Then, students’ use and exposure should be considered together with personal and societal factors in order to deal with it properly.

Findings clearly indicate that, regardless of their smoking status, more females than males have environmentally and socially sensitive opinions. One of the causes of the differences between the genders can be influence of the last remnants of traditional culture that downgrade and disgrace women who smoke cigarettes. We could hardly see women smoking cigarettes in 1960s in Turkey, but now, we see women smoking in everywhere. This means that the traditional culture on smoking has been waning. The differences on smoking status and opinions between females in private university and state university are also indicators of such culture change. Culture of smoking promotes private consumption by attaching it personal and exhibitionist values and gratifications that include irrational, illogical and dangerous elements. It is the part of misconstrued individualism that cultivates feelings and ideas that bestow relatively less care about others and environment and more care about one’s own personal choices, habits, needs and smoking practices. It is also a kind of change that first alienates people from each other and then bonds them through the cultural practices of conspicuous consumption: You gain your personal and interpersonal worth through the extend you own and use material things. Thus, brandishing an expensive cigarette package in a public place means marketing yourself and gaining your value. Such factors indicate that elimination of cigarette from human life requires diligent effort and pervasive programs and campaigns that encompass every sphere of daily life activities.

The present study supports the findings of previous studies that found out positive relationship between years at school and an increased rate of smoking [29, 31]. It also supports the conclusion of other studies that university students start smoking rather than quitting during their time in school [10–11]. Other important finding of the study is that more females start smoking as years pass in school. Such results suggest that it is necessary to design longitudinal studies to find out the detailed nature of changes in smoking at school.

The findings on the hypotheses # 3 and # 4 suggest that there are serious problems in formal schooling (and informal education) in educating rational, aware, sensitive, conscientious and environmentally and socially responsible citizens. Another suggestion is that establishing positive relations between educational level and variables like smoking, social consciousness, awareness, responsible behavior, rational decision making and rational behavior should be reconsidered as well as the causes of the failure of educational system. Furthermore, the interdeterminacy between the logic/rationalism and dominant cultural practices should be reevaluated in terms of perpetuation/ elimination of illogical (or reverse-logical) and irrational reasoning and behavior.

The difference between the private and state universities is an intricate finding because the students of the private universities have higher socio-economic status (SES). The future studies should provide a logical explanation for these differences.

Another implication of findings is that smokers’ opinions seem to be influenced by self-justification of their own daily practices that is the integrated part of the mind and behavior management practices (e.g. ads and promotions) of the tobacco and allied industries, and the prevailing cultural structure.

The student complaints and unstructured observations during the data collections reveal that there is a serious lack of anti-smoking policies in the universities. Some studies [4, 12, 13, 39–41] indicate that the lack of proper policies is most likely to contribute to a college environment that encourages smoking rather than helping students avoid smoking. In Turkey, many college students begin to smoke regularly during college, and only few of them stop smoking. Therefore, there is an urgent need to transform this environment by appropriate policies, programs and campaigns. For example, students who are occasional smokers may need programs to prevent them from becoming regular, nicotine-dependent smokers. College health centers can be an important source of assistance in quitting by providing smoking cessation programs and motivating students to take advantage of the programs.

Studies found that early university years are important to carry out anti-smoking activities for preventing students from starting smoking [28]. Reducing the visibility of tobacco use also discourage students from starting to smoke and make quitting easier [32]. Furthermore, some studies found that increased fines and excise taxes are the most effective intervention to reduce smoking [42]. Similar findings of other studies and findings of the present study reveal that the universities should enforce smoking bans throughout the campus, and forbid the sale, advertisement and promotion of cigarette at the university. A smoke-free university environment policy discourages smoking initiation, help smokers who are trying to quit, and protect nonsmokers from exposure to cigarette smoke.

The previous and present research findings also suggest that interventions aiming at preventing student smoking through legal sanctions would not be appropriate for inducing this age group to quit smoking, unless there is proper enforcement and education. Studies show that beliefs and knowledge of college students regarding the consequences of smoking appear rather limited; hence, anti-smoking campaigns need to communicate more effectively the concept and sensitivity that each cigarette they smoke is doing them and others serious damage [43, 44].

The greatest challenges in Turkey (and probably in similar countries) are to have professional health educators and proper administrative culture in order to address tobacco use, develop tobacco cessation programs that attract students and encourage smoke-free mind and environment. Previous studies indicate that proper policies may help deter students from developing or continuing cigarette smoking habit [34]. In order to further the public health goal of reducing cigarette smoking among youth, it has become commonplace to implement prevention programs at schools in many countries. However, the followings are highly visible facts of everyday life that we live in Turkey: “Tobacco-free environment” policies and programs either do not exist or remain on paper in Turkish universities. No organization and NGO are vigorously involved in recommending and promoting cigarette control policies for universities. No organization or group of academicians vigorously recommends that universities should prohibit the sale, advertising, sampling, and distribution of tobacco products on their campuses. No authority decidedly recommends that smoking should be banned at all university-sponsored indoor and outdoor events. There is no comprehensive policy and intervention designed to discourage students and teachers smoking. There is no university categorically prohibits smoking in all buildings, including student cafeterias, and provide systematic education about tobacco use. Faculty and university administrators’ efforts to respond to the smoking are almost nonexistent. They establish some “cigarette commissions or committees” that do little or nothing. University administrations do not give proper importance to the smoking problem. They place a few “no smoking” signs, and provide no systematic education about the consequences of smoking. Some administrators and teachers, while smoking, chat with students and expressively defend smoking in front of them. Teachers get together in their offices, chat and gossip, while smoking and drinking tea or coffee. Students do the same at the student cafeteria.

The present study extends prior research by investigating opinions of college students regarding smoking and policies in the university environment. Additional studies using student, teachers and school administrators from different universities and national representative samples are needed. The researchers should concentrate on achieving international participation, fostering discussion and promoting cooperation among scientists, policy makers, smokers and nonsmokers, children and students across geographical and disciplinary boundaries, and providing fresh findings and recommendations on tobacco production, distribution and use. Local, national and international responses to the tobacco issue should be intensified. Universities throughout the world should play the leading role in dealing with this serious problem.

There are some limitations of the present study. It was designed to test hypotheses that required bivariate analyses. It will be more informative if future designs that require multivariate analyses are prepared in order to account for confounding variables. There is a probability of sampling bias despite the fact that the study used a large sample size. However, the distribution of demographic characteristics (gender, school status) of the student population in the sample can be considered representative, because they have close proximity with the distribution in the population. Cross-sectional rather than longitudinal data were collected because of the nature of the research design. Cross-sectional analysis puts limits on directional analysis and comments of relationships. Longitudinal studies are needed to find out trends like change in smoking behavior and opinions as years pass in school. We set our opinion measures as dichotomous variables, because we were only interested in the two dimensions of opinions. The issue of quantitative or multi-level measurements with causal hypotheses might be designs that future research should consider.

## Figures and Tables

**Table 1:** Distribution of responses by gender.

*Issues*		*Female %*	*Male %*	*Both %*	*Test results*	
Smoking in the cafeteria	Disapprove	57.3	49.7	53.6	*N= 3643*	*X^2^= 21.40*
	Approve	42.7	50.3	46.4	*df= 1*	*p= 0.000*
Smoking in the school hallways	Disapprove	73.2	63.5	68.6	*N= 3650*	*X^2^= 39.51*
	Approve	26.8	36.5	31.4	*df= 1*	*p= 0.000*
Smoking indoors despite the law	Disapprove	86.7	79.3	83.1	*N= 3636*	*X^2^= 36.14*
	Approve	13.3	20.7	16.9	*df= 1*	*p = 0.000*
Teachers’ smoking in the classrooms	Disapprove	85.0	74.7	80.1	*N= 3640*	*X^2^= 60.85*
	Approve	15.0	25.3	19.9	*df= 1*	*p = 0.000*
Teachers’ smoking office doors open	Disapprove	66.8	61.9	64.4	*N= 3641*	*X^2^= 9.44*
	Approve	33.2	38.1	35.6	*df=1*	*p = 0.002*
School policy not enforcing the law	Disapprove	82.0	69.2	75.8	*N= 3631*	*X^2^= 82.15*
	Approve	18.0	30.8	24.2	*df=1*	*p = 0.000*
Smoking as freedom of choice	Disapprove	77.9	68.7	73.5	*N = 3632*	*X^2^= 38.86*
	Approve	22.1	31.3	26.5	*df = 1*	*p = 0.000*

**Table 2:** Distribution of responses according to cigarette use.

*Issues*		*Do you smoke cigarettes?*				*Test results*	
		*Yes %*	*Sometimes %*	*No %*	*All %*		
Smoking in the cafeteria	Disapprove	17.3	48.5	78.5	53.6	*N= 3649*	*X^2^= 1118.7*
	Approve	82.7	51.5	21.5	46.4	*df= 2*	*p= 0.001*
Smoking in the school hallways	Disapprove	35.5	69.2	89.8	68.6	*N= 3647*	*X^2^= 1015.5*
	Approve	64.5	30.8	10.2	31.4	*df=2*	*p = 0.000*
Smoking indoors despite the law	Disapprove	63.0	85.8	95.3	83.1	*N= 3633*	*X^2^= 554.4*
	Approve	37.0	14.2	4.7	16.9	*df=2*	*p= 0.000*
Teachers’ smoking in the classroom	Disapprove	60.8	79.2	92.8	80.1	*N= 3637*	*X^2^= 472.6*
	Approve	39.2	20.8	7.2	19.1	*df= 2*	*p= 0.000*
Teachers’ smoking office doors open	Disapprove	42.8	60.7	79.5	64.4	*N= 3638*	*X^2^= 1437.7*
	Approve	57.2	39.3	20.5	35.6	*df= 2*	*p= 0.000*
School policy not enforcing the law	Disapprove	48.1	76.2	93.6	75.9	*N= 3628*	*X^2^= 836.1*
	Approve	51.9	23.8	6.4	24.1	*df=2*	*p= 0.000*
Smoking as freedom of choice	Disapprove	59.4	67.9	84.2	73.5	*N= 3629*	*X^2^= 242.6*
	Approve	40.6	32.1	15.8	26.5	*df= 2*	*p= 0.000*

**Table 3:** School status by cigarette use *

*Years at school*		*Do you smoke cigarettes?*			*Row N*
		*Yes %*	*Sometimes %*	*No %*	
1	Male	37.3	14.4	48.3	485
	Female	23.6	14.1	62.3	554
	Both	30.0	14.3	55.7	1039
2	Male	37.9	13.9	48.2	375
	Female	28.2	13.7	58.1	475
	Both	32.4	13.8	53.8	850
3	Male	37.8	16.6	45.6	447
	Female	26.4	15.3	58.3	451
	Both	32.1	15.9	52.0	898
4 +	Male	45.4	15.1	39.5	438
	Female	34.6	16.1	49.3	416
	Both	40.2	15.6	44.2	854
All	Male	39.6	15.0	45.4	1745
	Female	27.8	14.7	57.4	1896
	Both	33.5	14.8	51.7	3641

* Partial distribution was provided only to give detailed information School classification by smoking: X2= 22.80 df= 6 p= 0.001

Females: X^2^ = 19.06 df= 6 p= 0.004

Males: X^2^ = 11.10 df= 6 p= 0.085

**Table 4:** Distribution of responses by the school status.

*Issues*		*Years at school*					*Test results*	
		*1 %*	*2 %*	*3 %*	*4 + %*	*All %*		
Smoking in the cafeteria	Disapprove	53.9	51.5	57.3	51.5	53.6	*N= 3631*	*X^2^= 8.03*
	Approve	46.1	48.5	42.7	48.5	46.4	*df= 3*	*p= 0.045*
Smoking in the school hallways	Disapprove	69.9	68.0	68.6	67.5	68.6	*N= 3638*	*X^2^= 1.40*
	Approve	30.1	32.0	31.4	32.5	31.4	*df= 3*	*p = 0.704*
Smoking indoors despite the law	Disapprove	85.1	83.0	83.9	79.9	83.1	*N= 3624*	*X^2^= 9.53*
	Approve	14.9	17.0	16.1	20.1	16.9	*df=3*	*p= 0.023*
Teachers’ smoking in the classroom	Disapprove	80.5	80.9	82.7	75.9	80.1	*N= 3628*	*X^2^= 13.14*
	Approve	19.5	19.1	17.3	24.1	19.9	*df= 3*	*p= 0.004*
Teachers’ smoking office doors open	Disapprove	65.4	64.1	65.7	62.0	64.4	*N= 3630*	*X^2^= 3.37*
	Approve	34.6	35.9	34.3	38.0	35.6	*df= 3*	*p= 0.338*
School policy not enforcing the law	Disapprove	77.3	75.9	76.5	73.6	75.9	*N= 3619*	*X^2^= 3.66*
	Approve	22.7	24.1	23.5	26.4	24.1	*df=3*	*p = 0.301*
Smoking asfreedom of choice	Disapprove	72.8	71.8	75.3	74.3	73.5	*N= 3620*	*X^2^= 3.38*
	Approve	27.2	28.2	24.7	25.7	26.5	*df= 3*	*p= 0.336*
